# Strategic Operational Redesign Improves Prior Authorization Access: A Validation Study

**DOI:** 10.14338/IJPT-23-00009.1

**Published:** 2023-11-24

**Authors:** Eric D. Brooks, Fantine Giap, Vincent Cassidy, Matthew S. Ning, Bradlee Robbert, Polly Redding, Matthew Palmer, L. Montreal Turner, William M. Mendenhall, Stuart Klein, Nancy P. Mendenhall

**Affiliations:** 1 Department of Radiation Oncology, University of Florida College of Medicine, Jacksonville, FL, USA; 2 Department of Radiation Oncology, The University of Texas MD Anderson Cancer Center, Houston, TX, USA; 3 University of Florida Health Proton Therapy Institute, Jacksonville, FL, USA; 4 Legion Healthcare Partners, LLC, Houston, TX, USA

**Keywords:** prior authorization, independent review organization, proton, radiation, approval

## Abstract

**Purpose:**

Obtaining prior authorization (PA) before treatment is becoming increasingly burdensome in oncology, especially in radiation oncology. Here, we describe the impact of a strategic novel operational PA redesign to shorten authorization time and to improve patient access to cancer care at a large United States academic proton therapy center. We ask whether such a redesign may be replicable and adoptable across oncology centers.

**Materials and Methods:**

Our PA redesign strategy was based on a 3-tiered approach. Specifically, we (1) held payors accountable to legally backed timelines, (2) leveraged expertise on insurance policies and practices, and (3) updated the submission, appeal writing, and planning procedures for PA. Metrics were compared at the following 3 time points: 6 months before, at phase-in, and at 6 months after intervention.

**Results:**

In analyzing the impact of improving PA access to care, the percentage of approvals for commercial proton beam therapy improved by an absolute 30.6% postintervention (*P* < .001). The proportion of commercially insured patients treated with proton beam therapy also increased by 6.2%, and the number of new starts rose by 11.7 patients/mo. Overall patient census increased by 13 patients/d. Median authorization time was 1 week, and 90% of surveyed providers reported reduced PA burden and improved patient care.

**Conclusion:**

This is the first validated, comprehensive operational strategy to improve access to cancer therapy while reducing the burden of PA. This novel approach may be helpful for addressing barriers to PA in medical and surgical oncology because the redesign is predicated on laws that regulate PA across disciplines.

## Introduction

The need for prior authorization (PA) by health insurance companies is increasingly recognized as a burden for providers and a barrier to patients seeking access to cancer care. Ostensibly, health insurers use PA to evaluate the appropriateness of a medical service to disincentivize costly procedures that do not provide comparable patient benefits [1, 2]. However, research on PA in radiation oncology—especially proton therapy—has shown no association between the approval of a recommended therapy and the type of tumor being treated, whether the patient is enrolled in research protocols, or the potential curative nature of the treatment, all factors historically considered sufficient for authorization. Thus, many proton therapy centers (PTC) view the PA process as ambiguous, arbitrarily subjective, and lacking in procedural justice for providing timely access to care [3, 4]. However, PA is not only a material liability to PTC operations but also to patient outcomes, as evidence indicates that each week of delay in treatment leads to a 1.2% to 3.2% increase in cancer death, a nonnegligible risk that could nullify the survival benefit of treatments that are eventually approved [5].

Recognizing these challenges and the increasing use of PA by payors over time, the University of Florida Health Proton Therapy Institute (UFHPTI) undertook a strategic redesign of its administrative PA process with the goals of reducing delays, denials, and costs while providing patients with more timely and appropriate access to care. This approach was based on a successful model described previously by another large cancer center [6] that used novel tactics to improve PA outcomes and burdens across several metrics [6]. We sought to validate that this approach could be replicated successfully at another large PTC.

## Materials and Methods

**Figure 1.**
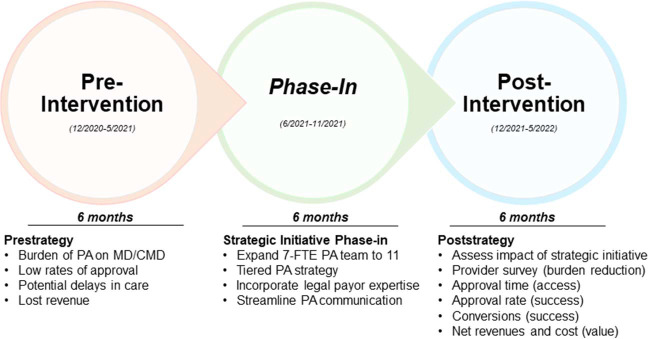
Strategic redesign for the prior authorization process over the 3 intervals in this study.

In June 2021, recognizing shifts in payor denial patterns and increased barriers to PA, UFHPTI executive leadership approached the developers of the prior strategy with the goal of redesigning the PA team and processing structure to minimize delays in PA and inappropriate denials (**Figure 1**). A 3-tiered approach to PA used successfully at another large PTC [6] was generated based on (1) a modified timeline for holding payors accountable to PA legally codified deadlines; (2) incorporating medical dosimetrists into the PA navigation process; and (3) updating the PA team’s knowledge of current commercial insurance policies and practices.

### Modified Timeline

The timelines and operations for the PA submission and appeals process at UFHPTI were modified as follows. First, we imposed a constrained timeline on payors to respond to initial submission, first appeal, second appeal, and independent review organization (IRO) review based on national and state mandates. The federal mandate is based on the Employee Retirement Income Security Act (ERISA) [7], which governs self-funded plans and requires payors to respond within 72 hours from the submission with their initial and subsequent determinations [7, 8].

Second, for traditional fully funded plans, state laws frequently mimic the ERISA or require payors to devise their own procedures and impose penalties if those PA procedures are not followed [9]. These self-imposed payor timelines are often similar to those under the ERISA. Part of the redesign of the PA process included a review of the state laws and plan-specific policies for clarity on how to hold payors accountable to timelines and patient rights for fully funded plans (eg, the PA statute in Florida [10]).

Third, for all plans (self- or fully funded) with a history of violated procedures or timelines or those with low approval rates, applicable state or federal laws were applied to skip internal payor appeals and move directly to IRO (eg, ERISA [7]). IRO is a unique method of alternative dispute resolution (IRO) required by many insurance companies to patients when internal appeals are believed to be denied inappropriately. This external independent review board evaluates the patient’s case and decides whether care is necessary and should be approved. IRO determinations are legally binding and can force coverage by the payor when a favorable determination is made (eg, ERISA [7]).

### Redesign of Prior Authorization Team

UFHPTI had an initial PA team consisting of 7 full-time-equivalent (FTE) members to shepherd coverage claims through the PA process. The redesign involved supplementing these 7 FTEs with another 4 individuals, including 2 new medical dosimetrists and 2 seasoned experts in PA navigation (11 total FTEs in the redesigned team; **Figure 1**). This strategy relieved much of the burden on the clinical dosimetry staff and providers, as the dosimetrists helped to prepare comparison treatment plans, coordinate peer-to-peer reviews, and write appeal letters. The initial PA team, the 4 new members, expert consultants, and UFHPTI executive leadership met twice weekly during the preimplementation and phase-in periods to strategize patient-specific PA approaches and ensure that the redesigned processes were being implemented. Results were also reviewed monthly and quarterly. A novel separate proprietary database was used to document each patient’s progress through the PA cycle, authorization status, and PA phase. Patients were also provided financial counseling and an overview of the PA process within 48 hours of their PTC consultation to enhance assurance and coordination of successful PA.

### Updating Team Knowledge

Three consulting individuals with payor and legal expertise or integral roles in devising and implementing the redesign at the other PTC [6] (EB, MP, and MT) guided the new redesign at UFHPTI. They provided consolidated weekly training sessions on state code and federal rules regulating PA timelines, IRO, strategies for appeal letters, and interfacing with payors or their contracted third-party vendor/administrators. Because the laws surrounding PA are continually being updated and strategies devised by payors evolve, the long-term success of a PA team depends on their having up-to-date knowledge of payor policies and practices. Providing team members with this information helped hold payors accountable to deadlines while empowering the PA team to use best practices or principled PA tactics.

### Analysis

Operational metrics were compared over three 6-month periods as follows: 6 months before the intervention (December 2020 to May 2021), 6 months after (June 2021 to November 2021), and 6 months after that (December 2021 to May 2022) (ie, preintervention, phase-in, and postintervention). The following metrics were analyzed and compared before versus after the intervention involved the patient populations affected by PA: (1) commercial-insurance approval rates for proton therapy; (2) IRO approval rate; (3) median time to an authorization decision; (4) daily census (on treatment) of commercially insured proton patients; (5) daily census of all proton patients; and (6) overall daily census and change in net revenue. A financial analysis was conducted to evaluate the value of the redesign on operations. This included a net present value (NPV; in US dollars) calculation over a 3-year period to represent a short-term valuation of the PA redesign investment based on the first year’s discounted free cash flow and assessment of the modified internal rate of return (MIRR, %). The NPV and MIRR used standard financial methods and incorporated excess net patient revenues resulting from additional approvals from the PA redesign over year 1, fixed and variable PA team costs, effort costs, and opportunity costs, as well as time period 0 costs. A constant discounted free cash flow model was subsequently used.

Pearson’s χ^2^ tests were used to compare the overall commercial proton approval rate after the redesign (and the proportion of appealed cases). For all analyses, the threshold for statistical significance was *P* = .05. A provider survey was furnished at 12 months postintervention to ascertain the redesign’s effect on clinical flow and reduction in PA burden.

## Results

Changes in operational metrics over the 3 intervals are shown in **Table 1**. The overall PBT approval rate by commercial insurers was 63% before the redesign and increased to 93.6% in the postintervention period (*P* < .001), amounting to a 30.6% absolute increase in the approval rate overall. Similarly, the IRO approval rate began at 0% and increased by 11% during phase-in and another 10% by postintervention, for an absolute increase of 21% overall.

**Table 1.** Preintervention, phase-in, and postintervention results.

Metric	Pre (12/20 to 05/21)	Phase-in (06/21 to 11/21)	Post (12/21 to 05/22)	Phase-in difference (preintervention to phase-in)	Overall difference (preintervention to postintervention)
Commercial proton approval rate, %	63	81.4	93.6	+18.4	+30.6
IRO approval rate, %	0	11	21	+11	+21
Median authorization time, workdays	N/A	7.1	6.9	N/A	N/A
Commercial proton new starts, mo	16.1	20.3	25	+4.2	+8.9
Commercial proton patients, %	36	36.3	42.2	+0.3	+6.2
Proton patient census, n	65.2	79.8	81.7	+14.6	+16.5
Daily census, n	82.3	93.7	95.3	+11.4	+13
Proton, %	79.3	85.1	85.7	+5.8	+6.4

**Abbreviations:** IRO, independent review organization; N/A, not applicable.

**Figure 2.**
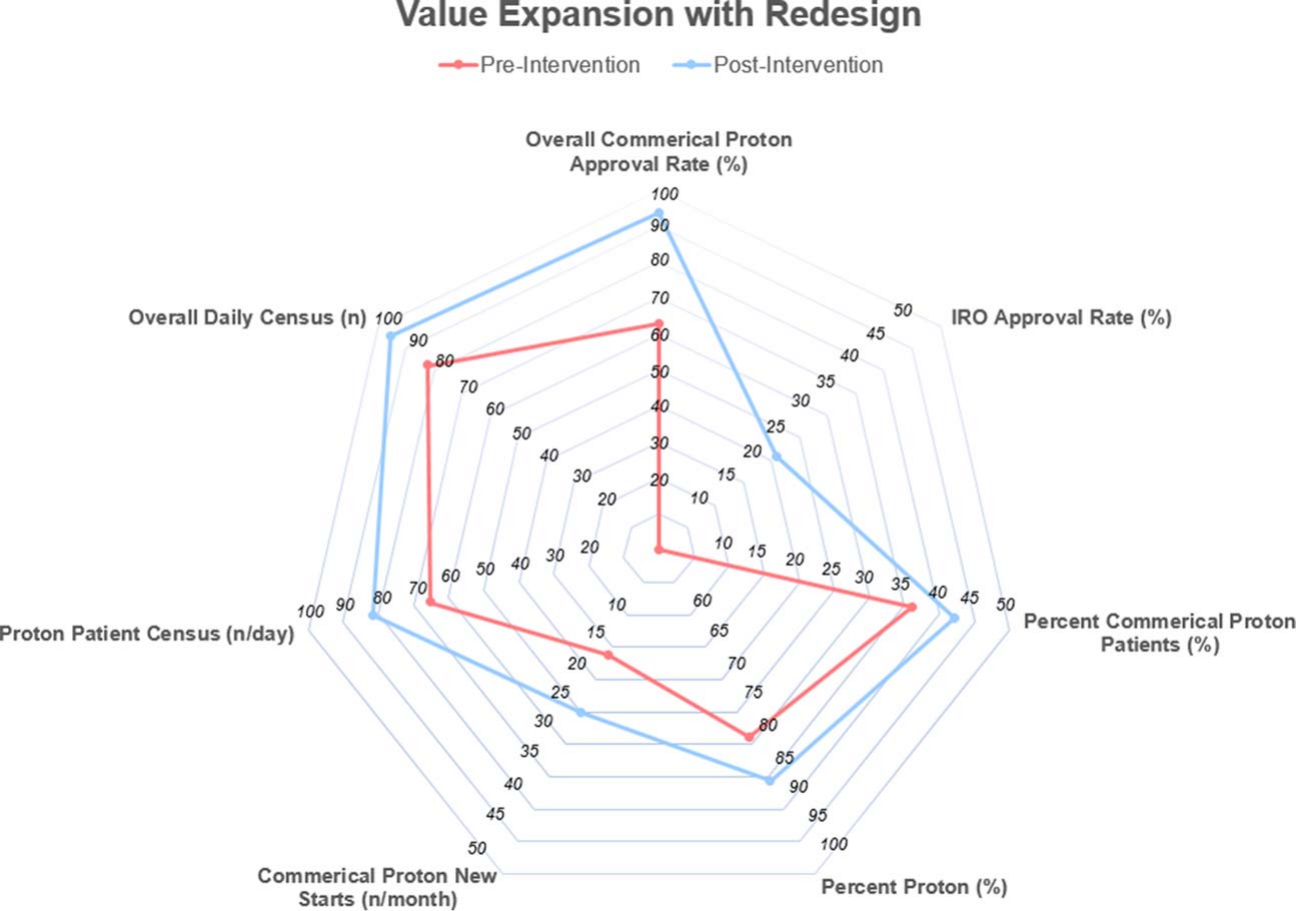
Radar plot showing the value-add of the prior authorization redesign to multiple operational metrics.

The number of new commercially insured proton starts, daily proton patient census, percentage of all patients receiving proton therapy, and percentage of commercially insured patients receiving proton therapy increased after the redesign. Specifically, the number of commercially insured proton new starts increased by 8.9 patients/mo; the daily census of all patients receiving proton therapy increased by 16.5 patients; and the percentage of patients receiving proton therapy (as opposed to photon or other types of radiation therapy) increased by 6.4%. Of note, the percentage of commercially insured patients receiving proton therapy increased by 6.2% (**Figure 2**). The median time to final authorization determination was 7.1 days after the redesign.

Analysis of financial metrics showed that the PA strategy yielded an NPV of 7- to 8-figures (USD) and an MIRR of 197%.

Last, a provider survey was sent to all 10 UFHPTI treating physicians 1 year after the redesign (**Table 2**). Of 9 physicians (90%) who responded, all reported that the intervention reduced or substantially eliminated the PA burden. Free-text feedback mentioned that providers had fewer letters of medical necessity, fewer peer-to-peer calls, and more timely approvals. Others noted that the redesign increased their confidence in prescribing and discussing proton therapy with patients without fear that appropriate treatment would be denied.

**Table 2.** Postintervention redesign effect—provider survey responses (N = 10; 90% response rate).

Question	Response
Has the redesign impacted your prior authorization workload (eg, LOMN writing, comparison plan design and review, P2P)?	90% say the redesign substantially reduced or eliminated PA burden
Has the redesign affected your perceived number of patients being approved for care?	90% say yes or possibly increased
How would you rate PA workflow and structure with the redesign compared with before?	90% say the redesign is minimally (10%), moderately (20%), or substantially/unequivocally better (60%); 10% say no change observed
How would you rate PA communication with administration and patients following the redesign?	75% say communication is minimally (25%), moderately (25%), or substantially (25%) better
Free-text feedback responses about the effects of the operational redesign on PA burden (2 text boxes allowed for free-text feedback).	“Overall, very happy with this [redesign].” “Nearly completely relief from all PA burden [and] less stress over the PA process.” “Reduced unnecessary paperwork (LOMN, dosimetry comparisons), [the redesign] increased my confidence to inform patients of the value of proton (I’m not as worried that they won’t be approved and then have to settle for IMRT or another modality).” “Less peer review calls.” “Fewer peer-to-peer discussions, faster answers.” “I am less involved in the authorization process [and] I suspect more patients are receiving authorization.” “[The redesign] has not impacted what I do in any way, positive or negative.” “I have not needed to write a LOMN since [we’ve done the redesign]! Also, I have spent much less time on comparison plans.” “[There is] less paperwork and peer to peers and easier approval process; Higher patient census.” “[The redesign] greatly reduced the peer-to-peer burden; increased and swifter authorization for protons.” “This is a good [redesign]; I have been impressed so far.” “[After the redesign, I have] no letter of medical necessity letters; no peer to peers; seemingly higher insurance approval rate.” “This has been a definite improvement in our PA process and has decreased the physician burden.”

**Abbreviations:** LOMN, letter of medical necessity; P2P, peer to peer; PA, prior authorization; IMRT, intensity-modulated radiation therapy.

## Discussion

To our knowledge, this is the first study to validate a redesigned PA process at an independent, university-based proton therapy facility. This novel strategy, which included restructuring an in-house PA team to include clinical medical dosimetrists, was initially piloted at another large academic proton center [6], where it was found to improve care delivery by reducing the median time to successful appeal from 30 to 18 days and by increasing the total number of overturned denials by 56%, both of which led to more patients being approved for care [6]. Using a similar approach, our PTC also saw sustained improvement in PA approvals and appeals by commercial insurers. Of note, our median time to authorization after the redesign was 6 to 7 days, a considerable improvement over the median of 18 days in the initial pilot study [6]. This discrepancy could be explained by knowledge gained from the initial operational redesign, overlap in key personnel between the pilot study and this one (EB and MP), or other factors. Nevertheless, this important study validated the use of a quality improvement approach within health care research and the role of operational studies in defining metrics that are important to improving access to high-quality care.

Although PA intends to limit excessive or unjustifiable therapies and protect patients from receiving nonevidence-based care, it also represents a significant barrier to necessary treatment, particularly in radiation oncology, because of outdated policies, not peer-reviewed, or inconsistently applied [4]. Critics of PA claim that it delays appropriate care, puts an undue administrative burden on physician practices, and leads to unpredictable decisions [11]. Our own experience with proton therapy for certain cancers shows that approval rates that were initially low eventually became high after a lengthy and arduous PA navigation process [12], raising the question of whether some mandates for PA by payors are warranted in the first place [3, 13]. A study published in 2019 reported that a simple PA can delay treatment starts by an average of 3 weeks, but those requiring an appeal can take up to 4 months [3]. Young, working-aged adults are the most likely to experience initial denial and longer wait times [14, 15]. Factors influencing initial PA approval and successfully appealed denials are often related to insurance type rather than clinical or treatment optimization factors [12, 13, 16]. All this highlights the burden on caregivers and patients and the need to address the PA process.

We found that our intervention substantially improved approval rates for proton therapy by commercial insurers. We attribute this 30.6% improvement to several elements of the redesigned processes. Chief among these elements is our robust operational structure based on clear statute-based timelines by which PA team members could unambiguously and explicitly hold payors accountable for rendering determinations. This structure not only fostered rapid decision times but also allowed data-driven decision-making to address PA issues in near real-time. For example, if monthly analyses revealed that certain payors were delaying determinations, then leadership, managed care directors, and other stakeholders could interface with the payors directly to enforce appropriate timeline compliance. At the very least, this tracking strengthened the arguments for approval at the appeal and IRO levels when violations were encountered; at best, it facilitated the pursuit of appeals within the shortest possible time to ensure no delays in treatment starts.

The second element of success was providing our internal PA team with the requisite knowledge of up-to-date payor policies and practices to improve the navigation of the entire process from the time of initial submission through potential IRO. Understanding how to optimize PA submissions through insights into payor processing and how best to structure an effective submission letter increased the number of approvals that did not need appeals. When an appeal was needed, the requisite clinical expertise by medical dosimetrists and trained PA team members allowed persuasive arguments, personalized for each patient, with the best odds for overturning denials.

The improved approval rate in this study ultimately manifested as meaningful improvements in patient access, as reflected by increases in proton new starts, the mix of commercially insured proton patients as a percent of total patient volume or census, and overall daily proton patients treated. Some of these benefits were recognized at 6 or 12 months, perhaps because of a lag time between insurance approval and proton therapy for cancers that require (neo)adjuvant treatment (eg, prostate or breast cancer).

From a value perspective, our analysis showed an impressive NPV of more than 7-figures and an MIRR of 197%. Unlike the original pilot study, we did not replace any PA team members or reduce the number of FTEs in that team. Although doing so led to cost savings in the pilot study [6], we invested in expanding the original 7-person PA team to include another 4 team members with the requisite expertise and trained the entire team, the cost of which was incorporated in the NPV and MIRR analysis. Despite these additional costs, plus the cost of leadership time for structuring and maintaining the redesign, the added value of our redesign was high. Moreover, our findings indicate that other centers can also achieve increased patient access and operations without the burden of completely overhauling existing staffing structures. However, the reduction in the burden of the PA process on the providers is another substantial benefit, both regarding administrative burden and in alleviating the declining enthusiasm for the specialty due to PA barriers to best practice, which has been reported as early as during training [17].

Another aspect of the success of our redesign is its generalizability to other US centers and specialties because the framework of our timeline is based on federal and state laws that would apply in any applicable US jurisdiction. For example, medical or surgical oncology practices could recruit advanced practice nurses to their PA teams who would have the clinical expertise for writing letters of medical necessity and drafting appeals; this would also lift the administrative burden of PA on providers in those specialties. Overall, our results indicate that this strategy can be extended beyond radiation oncology to medical and surgical oncology services because the redesign is predicated on laws and procedures that regulate PA for health services across disciplines, which is one of the most important effects of our findings.

However, crucial future directions for PA reform for proton therapy and other advanced treatment modalities in oncology include practice-level adoption of operational strategies to improve immediate access (as described here), as well as guidance on legislation to modify the regulation of PA with policymakers to prevent long-term detrimental effects on patient care. Reforming PA regulation may be necessary to achieve congruence between evidence-based national guideline recommendations [18] and payor policies and to prevent systematic delays and abandonment of care caused by inappropriate PA restrictions. In addition, direct collaboration with appropriate stakeholders, such as payors and employers, may improve patient access and reduce administrative burden without the overuse of proton beam therapy or other advanced care services, as was shown in a report in which proton coverage was adopted by statewide self-funded employer [19].

Our study had several important limitations. First, the improvement from the phase-in through postintervention periods may have been influenced by COVID-19 surges during the study. Although this would not directly affect approval rate metrics (the focus of the redesign), it could well have affected patient census. Similar COVID-19 spikes were experienced at our center’s location (Duval County, FL) during the preintervention and phase-in periods and, to a lesser extent, during postintervention. Despite these spikes, the trends seen in census improvement illustrate that the census benefits were likely related to redesign effects. Second, some benefits could have resulted from changes in payor policy, proton coverage expansion, disease site, or payor mix. However, no substantial changes were noted, and a review of the PA policies instead tended to become more restrictive over time [12, 17, 20]. Third, the time to final determination before the intervention was not available for comparison, mostly because of the lack of rigorous monitoring of PA metrics. However, the average 6 to 7 days until final determination at postintervention was significantly shorter than the 18-day average time to appeal after the intervention in the original pilot study. Fourth, redesign implementation can require a 6- to 8-month phase-in, during which staff must adapt to new roles, duties, team structures, and culture. Ensuring clear communication, holding regular team “huddles,” outlining goals, and offering the training required for new skill development are all imperative and should be factored into any valuation of the redesign, as we did here by accounting for FTE effort of leadership in team integration during period 0 in our MIRR and NPV modeling.

In closing, our redesign of the PA process confirmed that quality improvement efforts and operational research on factors related to initial PA approval and successfully appealed denials can produce practical data allowing practices to successfully navigate PA, thereby creating increased access to appropriate and justified care.

## References

[1] Schwartz AL Brennan TA Verbrugge DJ Newhouse JP Measuring the scope of prior authorization policies: applying private insurer rules to Medicare Part B *JAMA Health Forum* 2021 2 e210859. 35977311 10.1001/jamahealthforum.2021.0859 PMC8796979

[2] AHIP Prior Authorization: selectively used & evidence-based results of industry surveys November 2022. Accessed January 10, 2023. https://www.ahip.org/documents/Prior_Authorization_Survey_Infographic.pdf

[3] Gupta A Khan AJ Goyal S Millevoi R Elsebai N Jabbour SK Yue NJ Haffty BG Parikh RR Insurance approval for proton beam therapy and its impact on delays in treatment *Int J Radiat Oncol Biol Phys* 2019 104 714–23 30557673 10.1016/j.ijrobp.2018.12.021 PMC10915745

[4] Yu NY Sio TT Mohindra P Regine WF Miller RC Mahajan A Keole SR The insurance approval process for proton beam therapy must change: prior authorization is crippling access to appropriate health care *Int J Radiat Oncol Biol Phys* 2019 104 737–9 31204659 10.1016/j.ijrobp.2019.04.007

[5] Khorana AA Tullio K Elson P Pennell NA Grobmyer SR Kalady MF Raymond D Abraham J Klein EA Walsh RM Monteleone EE Wei W Hobbs B Bolwell BJ Time to initial cancer treatment in the United States and association with survival over time: An observational study *PLoS One* 2019 14 e0213209. 30822350 10.1371/journal.pone.0213209 PMC6396925

[6] Brooks ED Ning MS Palmer MB Gunn GB Frank SJ Shah AK Strategic operational redesign for successfully navigating prior authorization barriers at a large-volume proton therapy center *JCO Oncol Pract* 2020 16 e1067–77. 32639929 10.1200/JOP.19.00533 PMC8189610

[7] Employee Retirement Income Security Act 29 CFR § 2560.503-1 2016.

[8] Internal Claims and Appeals and External Review Processes 45 CFR § 147.136 2011.

[9] American Medical Association (AMA) 2021 prior authorization state law chart April 1, 2021. Accessed January 10, 2023. https://www.ama-assn.org/system/files/2021-04/pa-state-chart.pdf

[10] Fla Stat § 627.42392 (2017)

[11] Berg S What doctors wish patients knew about prior authorization AMA News Wire. July 29, 2022. Accessed January 10, 2023. https://www.ama-assn.org/practice-management/prior-authorization/what-doctors-wish-patients-knew-about-prior-authorization

[12] Mendenhall WM Brooks ED Smith S Morris CG Bryant CB Henderson RH Nichols RC Jr. McIntyre K Klein SL Mendenhall NP Insurance approval for definitive proton therapy for prostate cancer *Int J Part Ther* 2022 8 36–42 35127974 10.14338/IJPT-21-00002.1 PMC8768894

[13] Ning MS Gomez DR Shah AK Kim CR Palmer MB Thaker NG Grosshans DR Liao Z Chapman BV Brooks ED Tang C Rosenthal DI Garden AS Frank SJ Gunn GB The insurance approval process for proton radiation therapy: a significant barrier to patient care *Int J Radiat Oncol Biol Phys* 2019 104 724–33 30557675 10.1016/j.ijrobp.2018.12.019

[14] Bishop AJ Livingston JA Ning MS Valdez ID Wages CA McAleer MF Paulino AC Grosshans DR Woodhouse KD Tao R Roth ME Gunn GB McGovern SL Young adult populations face yet another barrier to care with insurers: limited access to proton therapy *Int J Radiat Oncol Biol Phys* 2021 110 1496–504 33677051 10.1016/j.ijrobp.2021.02.049 PMC8286292

[15] Ning MS Shah AK Smith GL Palmer MB Brooks ED Cantor S Huang D Gunn GB Frank SJ Insurance coverage gaps for proton therapy: vulnerable patient populations *Int J Radiat Oncol Biol Phys* 2019 105 E447–8

[16] Bekelman JE Asch DA Tochner Z Friedberg J Vaughn DJ Rash E Raksowski K Hahn SM Principles and reality of proton therapy treatment allocation *Int J Radiat Oncol Biol Phys* 2014 89 499–508 24798985 10.1016/j.ijrobp.2014.03.023 PMC4278661

[17] Kim H Srivastava A Gabani P Kim E Lee H Pedersen KS Oncology trainee perceptions of the prior authorization process: a national survey *Adv Radiat Oncol* 2022 7 100861. 35118213 10.1016/j.adro.2021.100861 PMC8792423

[18] Verma V Ludmir EB Mesko SM Brooks ED Augustyn A Milano MT Lin SH Chang JY Welsh JW Commercial insurance coverage of advanced radiation therapy techniques compared with American Society for Radiation Oncology model policies *Pract Radiat Oncol* 2020 10 324–9 31446147 10.1016/j.prro.2019.08.005

[19] Ning MS Palmer MB Shah AK Chambers LC Garlock LB Melson BB Frank SJ Three-Year Results of a Prospective statewide insurance coverage pilot for proton therapy: stakeholder collaboration improves patient access to care *JCO Oncol Pract* 2020 16 e966–76. 32302271 10.1200/JOP.19.00437 PMC8462618

[20] Waddle M Miller R The Economics of Particle Therapy In: Waddle M Miller R , eds. *Principles and Practice of Particle Therapy*; Hoboken, NJ Wiley-Blackwell 2022: 165–75

